# Cryptic *Plasmodium ovale* concurrent with mixed *Plasmodium falciparum* and *Plasmodium malariae* infection in two children from Central African Republic

**DOI:** 10.1186/s12936-017-1979-5

**Published:** 2017-08-15

**Authors:** Cynthia Bichara, Philippe Flahaut, Damien Costa, Anne-Lise Bienvenu, Stephane Picot, Gilles Gargala

**Affiliations:** 1 Department of Parasitology-Mycology, Rouen University Hospital, 76 000 Rouen, France; 2 Department of Pediatrics, Rouen University Hospital, 76 000 Rouen, France; 3 Malaria Research Unit, SMITh, ICBMS UMR 5246 CNRS, University Lyon 1, Lyon, France; 4 Institut de Parasitologie-Mycologie Médicale, Hospices Civils de Lyon, Lyon, France

**Keywords:** Malaria, *Plasmodium*, Cryptic, Diagnosis, Follow-up

## Abstract

**Background:**

Since several malaria parasite species are usually present in a particular area, co-infections with more than one species of *Plasmodium* are more likely to occur in humans infected in these areas. In many mixed infections, parasite densities of the cryptic species may be low and often not recognized in clinical practice.

**Case presentation:**

Two children (3 and 6 years old) adopted recently from Central African Republic were admitted to hospital because of intermittent fever. Thin blood smears stained with Giemsa showed *Plasmodium falciparum* and *Plasmodium malariae* co-infection for both children at admission. They were both treated with atovaquone-proguanil combination for 3 days. At day 7, both thin blood smears examination remained negative but at day 28, thin blood smear was positive for *P. malariae* trophozoites and for *Plasmodium ovale* for the girl and her brother, respectively. Samples collected at day 1 and day 28 were submitted to real-time PCR showing the presence of the three parasite species (*P. falciparum*, *P malariae* and *P. ovale*) in admission blood samples from the two children and only *P. ovale* at day 28.

**Conclusions:**

Twenty-eight days follow-up after treatment led to detection of a third parasite species in the blood of these two patients suggesting covert co-infection and a delayed appearance of one cryptic species following treatment. Concurrently infecting malaria species could be mutually suppressive, with *P. falciparum* tending to dominate other species. These observations provide more evidence that recommendations for treatment of imported malaria should take into account the risk of concurrent or cryptic infection with *Plasmodium* species. Clinicians and biologists should be aware of the underestimated frequency of mixed infections with cryptic species and of the importance of patient follow-up at day 28. Future guidelines should shed more light on the treatment of mixed infection and on the interest of using artemisinin-based combinations for *falciparum *and non-*falciparum *species.

## Background

Mixed-species malaria infections have received sporadic attention [1], and the trend toward malaria control in some endemic areas should reduce the occurrence of concurrent infection with more than one species of *Plasmodium*. However, diagnostic of these mixed-infections is rare in clinical practice, and probably underestimated by microscopy. Expert microscopists are uncommon in clinical laboratory, and distinguishing the species from young trophozoite forms of *Plasmodium* parasites is sometimes difficult [2]. The life-threatening *P. falciparum* is obviously taking most of the attention considering the potential consequences of a wrong diagnosis, and other species are sometimes scanned with less intensity. In case of mixed infection, the concurrent species is mostly below the threshold of microscopy. For example, the ratio of *Plasmodium malariae/P. falciparum* was described as extremely low in a study conducted in Ivory Coast and the protective effect of lengthy subclinical *P. malariae* infections was suspected [3]. Moreover, the liver stage of *Plasmodium vivax* and *Plasmodium ovale,* which are responsible for relapses, are not detectable [1]. The delayed appearance of a cryptic species after successful treatment of the first diagnosed species has been reported in area endemic for *P. falciparum* and *P. vivax* malaria [4].

It has been known from decades that antagonism between species may lead to a rapid decrease of circulating parasites, such as *P. malariae* when inoculated with *P. vivax* in an attempt to treat neurosyphilis [5].

Triple species infection are known, but remain rare in the literature. Two parallel cases of triple infections (*P. falciparum* and *P. malariae,* followed by *P. ovale* delayed infection) in two adopted children from Central African Republic are presented here to document the risk of late reappearance and the need for clinical and parasitological follow-up. These cases focused more light on the risk of treating each malaria species by drugs with different efficacy.

## Case report

Two children (3 and 6 years old) were admitted to hospital because of intermittent fever and suspected malaria. They have been adopted recently and came back from Central African Republic a week previously.

The 6-year-old boy presented with a temperature of 37.9 °C, a systolic murmur in mitral focus, and a hepatosplenomegaly. The haemoglobin level was 6.7 g/dL, the mean corpuscular volume was 65 fL, and the platelet count was 216 G/L. Biochemical tests showed a normal liver function, increased serum levels of lactate dehydrogenase (291 U/L, reference range 135–250 U/L) and C-reactive protein (73, reference value <5). A coagulation test result for the prothrombin time–international normalized ratio was slightly prolonged (14.7 s with a ratio at 67%, reference range 12.5 s with a ratio at 75–100%). An ultrasonographic abdominal examination showed hepatomegaly (107 mm thickness along the mammary line, reference value for this age 90 mm), and splenomegaly (115 mm, reference range 70–96 mm), and lack of deep lymph nodes. An ultrasonographic heart examination reported no abnormalities.

The 3-year-old girl, presented with a temperature of 37 °C, bilateral crackles predominant right, and a hepatosplenomegaly. The haemoglobin level was 6.3 g/dL, the mean corpuscular volume was 71.6 fL, and the platelet count was 138 g/L. Biochemical tests showed a normal liver function, increased serum levels of lactate dehydrogenase (379 U/L) and C-reactive protein (48 mg/mL). A coagulation test result for the prothrombin time–international normalized ratio was slightly normal. Ultrasonographic abdominal examination reported a splenomegaly (100 mm, and lack of deep lymph nodes.

Thin blood smears stained with Giemsa showed *P. falciparum* and *P. malariae* co-infection for both children (*P. falciparum* 1.3% + *P. malariae* 0.14% and *P. falciparum* 0.03% + *P. malariae* 0.15% for girl and boy, respectively). A rapid diagnostic test (Palutop4 +, AllDiag, Strasbourg, France) showed three positive bands for the control, *Plasmodium* spp. (pLDH), and *P. falciparum* (pHRPII) markers.

Both children received 1 adult co-formulated tablet of Malarone, containing 250 mg atovaquone and 100 mg proguanil hydrochloride, per day for 3 days. The tablets were administered during the meal without addition of fat. The boy weighed 19 kg, the girl weighed 14.5 kg and the administered weight-based dose of atovaquone/proguanil was 10/5 and 17/7 mg/kg/day for the boy and girl respectively. They did not vomit and no other adverse events were noted during treatment. Both children received iron syrup supplementation and paracetamol.

Almost complete parasitaemia clearance was obtained at day 3 (*P. falciparum* <0.01% + *P. malariae* <0.01% and *P. falciparum* 0% + *P. malariae* 0.02%, for girl and boy respectively). These figures are not uncommon because atovaquone-proguanil is known to act relatively slowly. Apyrexia was obtained at day 2 and patients were discharged on day 5 and did not return until scheduled appointments for control of parasitaemia on day 7.

At day 7, both thin blood smears examination remained negative, while few picnotic parasites were detected on thick blood smears. Surprisingly, at day 28, thin blood smear from the girl was positive and revealed <0.01% *P. malariae* trophozoites while *P. ovale* (<0.01%) was detected from her brother’s blood. To confirm the species identification, the samples collected at day 1 and day 28 were submitted to real-time PCR as previously described [6]. Molecular diagnosis demonstrated the presence of the three parasite species (*P. falciparum, P. malariae* and *P. ovale*) in D0 samples from both children, and only *P. ovale* at day 28.

Both children were still apyretic and didn’t show any symptoms of infection. Considering the suspected parasitological failure of the atovaquone-proguanil treatment against *P. ovale*, patients were given a total dose of 25 mg/kg of chloroquine over 3 days. Both children were not glucose-6-phosphate dehydrogenase-deficient, but primaquine was not used to prevent relapses. Clinical and parasitological follow-up was performed and total parasite clearance was assessed by thin and thick blood smears at day 42, showing the absence of recurrence. Both children had non-haemolytic (normal haptoglobin), microcytic anaemia at admission to hospital and were found to have iron deficiency, which was successfully treated after 3 months of iron supplementation. Echocardiography examinations revealed that boy had an inorganic heart murmur. Gastrointestinal helminths were not found after 3 stool examinations for ova and parasites. Cysts of *Entamoeba coli* and *Chilomastix mesnili* were found in the boy’s stools, who was treated with secnidazole. Patients were then lost to follow-up.

## Conclusion

Six different malaria species are known to infect humans: *P. falciparum, P. vivax, P. malariae, P. ovale wallikeri, P. ovale curtisi* and *P. knowlesi* [7]. Routine microscopical examination of thick and/or thin blood smears stained with Giemsa allows discrimination of four of these species, since *P. ovale wallikeri* is not distinguishable from *P. ovale curtisi*, and *P. knowlesi* is impossible to distinguish from *P. malariae*. Most of the rapid diagnostic tests available on the market showed lower performance for *P. vivax* (70.7% panel detection score) than for *P. falciparum* (85% panel detection score) in the round 6 of WHO [8]. *Plasmodium ovale* and *P. malariae* RDTs have not been evaluated so far. Molecular tests are the only methods allowing species identification with a good accuracy.

Mixed, or concurrent infections, are frequently reported in epidemiological studies conducted in endemic areas and probably underdiagnosed at an individual level. It is known that several *Anopheles* species, are able to carry more than one species, including the four main species for *Anopheles gambiae* [9]. There is no definitive evidence of a clinical or parasitological impact of mixed infections in humans. *Plasmodium vivax* is supposed to reduce the severity of *P. falciparum* infection; *P. falciparum* reduces the availability of red blood cells for *P. vivax* infection, and a theory of species-transcendenting density-dependant (STDD) regulation of multiple malaria infection has been proposed [10, 11].

There is no evidence that drugs used to treat the dominant specie may be ineffective against the cryptic species. Since ACTs are mainly recommended to treat *falciparum *malaria, and chloroquine to treat non-*falciparum *malaria, under-diagnosis of mixed infection may lead to inappropriate treatment of patients. In countries with chloroquine-resistant *P. vivax*, ACT is the treatment policy for non-*falciparum *infections. ACT is also recommended treatment of mixed infections [12]. In the two cases presented here, *P. falciparum* and *P. malariae* co-infection was successfully treated with atovaquone-proguanil, while asymptomatic *P. ovale* was detected at day 28. Unfortunately, it was not possible to know if the recurrence of *P. ovale* was due to drug resistance or relapse of liver stages, and cytochrome b gene mutation was not investigated due to low amount of samples. A plausible cause of this late therapeutic failure is the relatively insufficient dosage due to increased oral clearance of atovaquone in pediatric patients. However, failure to atovaquone-proguanil prophylaxis has been reported in travelers presenting *P. ovale* infection [13].

These observations provide more evidence that recommendations for treatment of imported malaria may take into account the risk of concurrent or cryptic infection with *Plasmodium *species presenting different sensitivity to drugs. Considering that information on drug resistance of non-*falciparum *species is scarce, clinician and biologists should take particular attention to patient follow-up at day 28.

## Abbreviations

fL: fento-liter
STDD: species-transcendenting density-dependant
